# Exploring the Potential of Spherical Harmonics and PCVM for Compounds Activity Prediction

**DOI:** 10.3390/ijms20092175

**Published:** 2019-05-02

**Authors:** Magdalena Wiercioch

**Affiliations:** Jagiellonian University, Faculty of Physics, Astronomy and Applied Computer Science, S. Łojasiewicza Street 11, 30-348 Kraków, Poland; mgkwiercioch@gmail.com

**Keywords:** representation learning, cheminformatics, molecular representation, G protein-coupled receptors, machine learning, molecular activity predictions

## Abstract

Biologically active chemical compounds may provide remedies for several diseases. Meanwhile, Machine Learning techniques applied to Drug Discovery, which are cheaper and faster than wet-lab experiments, have the capability to more effectively identify molecules with the expected pharmacological activity. Therefore, it is urgent and essential to develop more representative descriptors and reliable classification methods to accurately predict molecular activity. In this paper, we investigate the potential of a novel representation based on Spherical Harmonics fed into Probabilistic Classification Vector Machines classifier, namely SHPCVM, to compound the activity prediction task. We make use of representation learning to acquire the features which describe the molecules as precise as possible. To verify the performance of SHPCVM ten-fold cross-validation tests are performed on twenty-one G protein-coupled receptors (GPCRs). Experimental outcomes (accuracy of 0.86) assessed by the classification accuracy, precision, recall, Matthews’ Correlation Coefficient and Cohen’s kappa reveal that using our Spherical Harmonics-based representation which is relatively short and Probabilistic Classification Vector Machines can achieve very satisfactory performance results for GPCRs.

## 1. Introduction

Rational drug discovery aims at the identification of ligands that act on single or multiple drug targets [1,2,3]. The process is usually performed by research which is focused on developing methods and tools for understanding chemical space. In order to find the desired candidates, several computational approaches are required which enable to predict drug-like properties.

Take for instance virtual screening [4], which has its roots in cheminformatics and performs the rapid in silico assessment of large libraries of chemical structures to identify those most likely to bind to a drug target. Recently, one may observe the success and possible new opportunities with regards to ligand-based virtual screening [5]. In this modern era of computational technological advancement, machine learning has been extensively applied to predict the activity of new candidate compounds. Willett et al. proposed a binary kernel discrimination approach [6]. The multidimensional analysis of classification performance of compounds were performed by Smusz et al. [7]. The Bayesian belief network was adopted by Nidhi et al. [8] and Xia et al. [9]. A lot of promising prediction results by adopting Support Vector Machines were obtained by Buchwald et al. [10], Bruce et al. [11] Czarnecki et al. [12], Rataj et al. [13], and Zhang et al. [14]. Liu et al. have constructed ensembles to identify Piwi-Interacting RNAs [15].

However, the success of applied machine learning methods depends on the molecular structure representation employed, also known as the molecular descriptors [16]. Thus, the main challenge is to devise representations of molecules that are both complete and concise to enable to reduce the number of calculations that are needed to predict the properties [17]. There has been a flood of interesting approaches to represent molecules [18]. For instance, classical QSAR (Quantitative Structure-Activity Relationships) methodologies [19] have given their contribution [20,21,22,23]. Lozano et al. identified molecular features responsible for the antileishmanial activity of 61 adenosine analogues acting as inhibitors of the enzyme glyceraldehyde 3-phosphate dehydrogenase of *Leishmania mexicana* (LmGAPDH) [24]. Adeniji et al. made a great effort to develop a model that relates the structures of 50 compounds to their activities against *M. tuberculosis* [25]. In [26], the authors propose new amino acid descriptors which should result in more readily interpretable models for the enzyme activity of proteins. Limitations of QSARs were addressed by Tong et al. [27]. Ghasemi et al. analyzed neural network and deep-learning algorithms used in QSAR studies [28]. Lately, Consonni et al. introduced a new metric to estimate the model predictive ability of QSARs [29].

As was previously mentioned, representation learning, a part of machine learning, also serves to provide new descriptors [30]. Kuroda presented a novel descriptor based on atom-pair properties [31]. Śmieja et al. investigated a new approach for fingerprint hybridization and reduction [32]. A molecular descriptor obtained by translating equivalent chemical representations was developed by Winter et al. [33]. Wang et al. explored protein-protein interactions prediction using Zernike moments descriptor [34]. Recently, the feature representation problem in bioinformatics was analyzed by Li et al. [35]. In [36] the authors strive to provide a novel local conjoint triad feature representation. Additionally, recent studies address the challenges faced in developing molecular descriptors and tools to drug design targeting GPCRs [37,38].

At the same time, G protein-coupled receptors (GPCRs) are part of a large group of signaling proteins that mediate cellular responses to most metabolites, hormones, cytokines and neurotransmitters. For this reason, GPCRs have been extensively explored as important drug targets [39]. Research indicates GPCRs are the targets of nearly 35% of all drugs approved by the US Food and Drug Administration [40]. In the era of Computer-Aided Drug Design (CADD) machine learning techniques can be used to discover active ligands and predict the activity of molecules.

**Figure 1:**
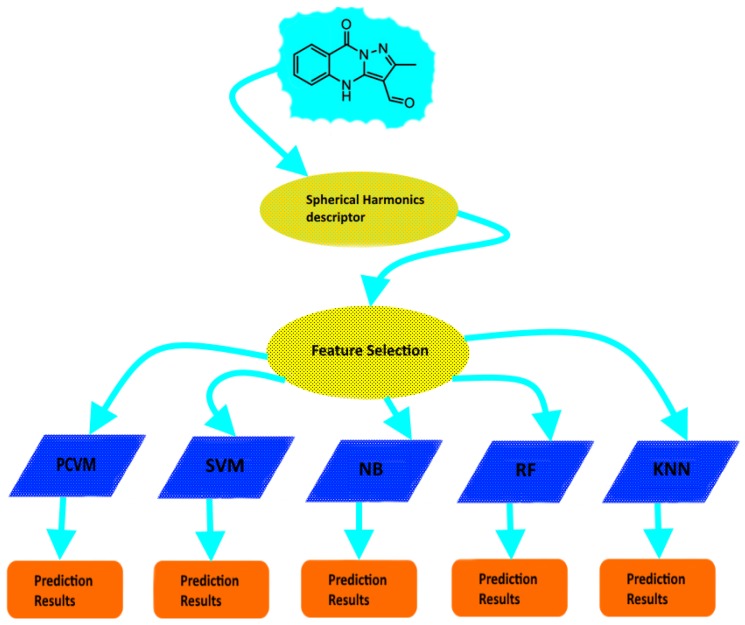
Flowchart of research methodology.

In view of the above, in this study we focused on improving molecular activity prediction. We introduce a novel methodology that involves Probabilistic Classification Vector Machines (PCVM) and Spherical Harmonics-based descriptor which we call SHPCVM. Previous work has shown that PCVM plays a prominent role in prediction-based processes [34]. Additionally, Spherical Harmonics have been successfully applied to cheminformatics [41,42]. Nevertheless, the key principle of our Spherical Harmonics-based approach is not the usage of Spherical Harmonics themselves but the fact that our technique makes use of our feature selection strategy, namely Minimum Redundancy and Maximum Relevance (MRMR) that enables obtaining only representative features. Although previous studies also indicate a few attempts have been made to employ feature selection methodologies to cheminformatics and bioinformatics [43,44,45], our methodology is novel. Finally, the vector representation that we get is relatively short and more discriminative. The presented method was applied to 21 GPCR datasets. In particular, the computer experiments included the comparison with both competitive classifiers (Naïve Bayes, *K* Nearest Neighbours, Support Vector Machines and Random Forests) and other representations (MOE and Connectivity descriptor). The results suggest that SHPCVM is superior to other approaches. Therefore, this technique is adequate for molecular prediction and may be further explored. Flowchart of our research methodology is shown in Figure 1.

The rest of this paper is organized as follows. Section 2 introduces the evaluation measures used in the computer experiments, describes the architecture and demonstrates the results with a discussion on influence of our methodology on prediction ability. The third section studies the datasets and explains all applied methods. Section 4 summarizes the work presented in this paper.

## 2. Results And Discussion

In this section we present the evaluation measures employed for performance comparison. Then we analyze and discuss the experimental results and compare our results with other approaches.

### 2.1. Evaluation Measure

We considered compound activity prediction as a binary classification task. Hence, a number of commonly used measures can be employed to evaluate its performance. These methods include accuracy (ACC), precision (PRE), recall (REC), the Matthews Correlation Coefficient (MCC) and the Cohen’s kappa (κ). They are listed in Table 1.

### 2.2. Experimental Design

In the study, the flowchart of SHPCVM is shown in Figure 1. More specifically, after getting the data the spherical harmonics-based descriptor is calculated. In order to obtain the optimal number of features, we perform feature selection process. Then the final molecular descriptor is used as input to train the PCVM classifier. We divided the datasets into training (80%) and test (20%) sets to carry out the computer experiments. Since cross-validation is a useful tool to select the appropriate model and tune a few parameters, ten-fold cross-validation was used for the training purposes. Finally, the performance of each classifier was evaluated on an external test set randomly selected from the original dataset (20%).

We used in-house Python code for features calculations and the scikit-learn package (http://scikit-learn.org/) for machine learning. 3D coordinates for the molecules were generated using 2D→3D structure generation routines included in the RDKit [49] and Open Babel [50] python packages. Both Connectivity descriptor and MOE-type features for each molecule were calculated by Python ChemoPy package [51].

### 2.3. Descriptor Insights

**Figure 2:**
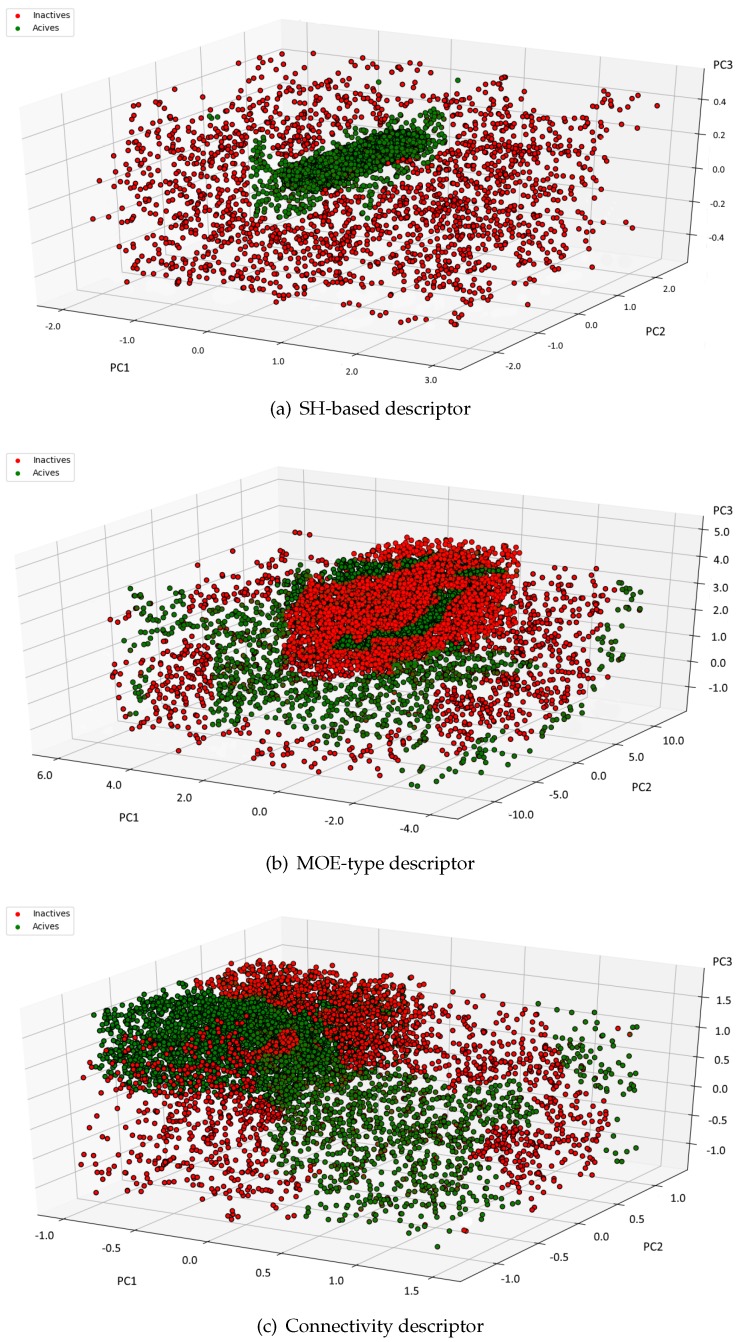
Scattergram of (**a**) Spherical Harmonics-based, (**b**) MOE-type and (**c**) Connectivity descriptor for both active and inactive compounds in P35372 dataset.

The main goal of any molecular descriptor is to achieve a mapping from the original space to another designed descriptor space. Since the new space usually has a smaller dimension, some information will be inevitably lost after the reduction. Thus, a perfect descriptor is supposed to preserve the core information. In our computer experiments we have examined whether the spherical harmonics-based descriptor meets the expectations. We have performed PCA [52] on 49 dimensional descriptor and analyzed the quality of the separation between active and inactive molecules. PCA is a well-known and widely used method that projects a dataset onto the directions that account for most of the variance in the dataset. Figure 2 shows the distribution of the active and inactive compounds in P35372 dataset after applying PCA to the 49 dimensional spherical harmonics-based descriptor, MOE—type and Connectivity descriptor, and choosing the top three principle components. One may notice that the biologically active compounds are gathered together.

On the other hand, the inactive compounds are spread out. Obviously, the active and inactive molecules are not completely separated. However, it is quite easy to notice some patterns and clusters of actives and inactives. The visual inspection suggests that the spherical harmonics-based descriptor preserves most of information to allow classification and can be further explored. Please note that the goal of this computer experiment was to ensure whether the information preserved by the descriptors may be enough to apply the representation to search for active compounds. If the descriptor was useless, the data would be randomly separated and none interesting patterns could be observed. Indeed, Figure 2 indicates the data described by spherical harmonics based descriptor is not linearly-separable but we did not expect it. Instead, we have found out the descriptor is a good tool to analyze the chemical space. What is more, to give an illustrative example Figure 2 shows the distribution of data for only 1 out of 21 sets included in the datasets. However, we have observed similar tendency in all datasets.

**Figure 3:**
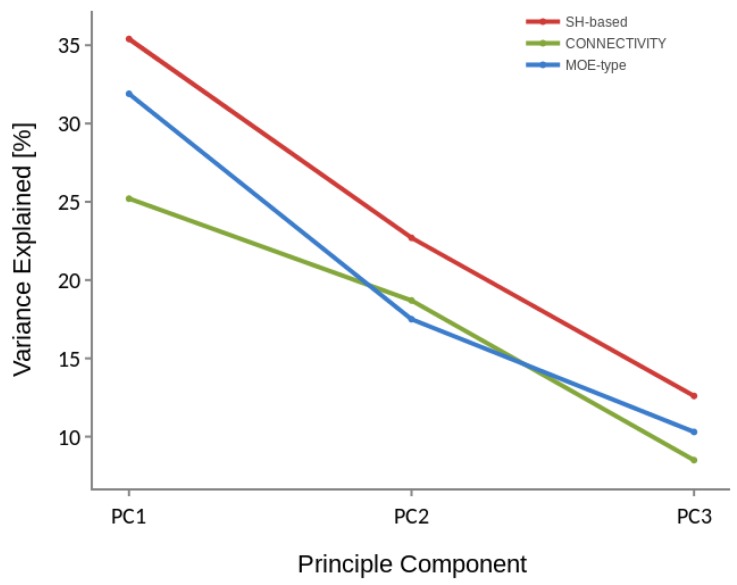
Three principal components ranked by the amount of variance they capture in P35372 dataset for Spherical Harmonics-based, MOE-type and Connectivity descriptor.

The results of PCA applied to P35372 dataset, i.e., the percentage of the variation explained by each principal component for three different descriptors are shown in Figure 3. It can be noticed that for Spherical Harmonics-based descriptor the top three principle components explain more than 70% of the variation of samples in the descriptor space. It suggests that the 3D spatial distribution illustrated in Figure 2 may, at least partially, reflect the real spatial distribution in the descriptor space. Moreover, the PCA results indicate the actives and inactives represented by the three descriptors (MOE, Connectivity and SH-based) are not linearly separable. Nevertheless, such data can still be classified correctly using some non-linear approaches.

### 2.4. Performance Evaluation

The purpose of the computer experiments presented in this subsection was three-fold. As the introductory computer experiments described in Section 2.3 have demonstrated, the spherical harmonics-based descriptor is a reliable descriptor to analyze the molecular space. For this reason, our first goal is to assess the ability of PCVM classifier with the spherical harmonics-based descriptor to predict biologically active compounds. Secondly, we aimed to compare the PCVM performance with SVM approach and another classifiers. Finally, we compared the prediction performance of PCVM as a representative classification method when different descriptors are used.

#### 2.4.1. PCVM Model with a Spherical Harmonics-Based Descriptor

After ten-fold cross-validation procedure, a performance estimate was obtained for each test dataset. The outcomes over the evaluation measures for PCVM and the molecules are shown in Table 2, Table 3, Table 4, Table 5 and Table 6. The results suggest that the proposed approach is valuable. We observed that ACC is more than 0.8 in the vast majority of cases. The minimum values for ACC, PRE, REC, MCC and κ are 0.742, 0.726, 0.752, 0.69, and 0.651 respectively.

The results illustrated in Table 2, Table 3, Table 4, Table 5 and Table 6 indicate that our approach has good discriminative capabilities for the molecular activity recognition. One may notice it is able to outperform representative models. The corresponding outcomes obtained by cross-validation on the training set are available as Supplementary Materials. Based on reported values, SHPCVM is indeed a robust approach. It appears the results can be replicated on unseen data.

A point to consider is the fact that our final representation is strictly dependent on the precision of 3D structure model. Consequently, for different conformations, we get different representation of the given molecule. Also, the quality of 3D structure is significant. Here, we want to stress that although the molecular activity is the joined effect of varied factors (physico-chemical and biochemical properties, among others), PCVM combined with the new shape-based representation is able to give good prediction outcomes. Our results again indicate that the choice of a proper set of features which describe the molecule may affect prediction performance. Furthermore, the choice of PCVM model as a classifier is meaningful as well. This fact is explored in the next computer experiments.

#### 2.4.2. SVM Model with a Spherical Harmonics-Based Descriptor

Inspired by the previously shown results we validated the performance of SVM [53] classifier and compared it with PCVM. Table 2, Table 3, Table 4, Table 5 and Table 6 display all five measures. They illustrate that the highest accuracy obtained by SVM was 0.826 for Q9Y5N1. Interestingly, PCVM achieved 0.862. Furthermore, the maximum values for ACC, PRE, REC, MCC and κ are 0.826, 0.849, 0.831, 0.753. and 0.741, respectively. The smallest accuracy rate is reported for P30542 and equals 0.712. For the other measures the minimum values for P30542 (PRE, REC, MCC, κ) are 0.696, 0.725, 0.654 and 0.615. It is worth noticing that for the same dataset PCVM yields 0.742, 0.726, 0.752, 0.691 and 0.651 for ACC, PRE, REC, MCC and κ which is better than SVM.

**Figure 4:**
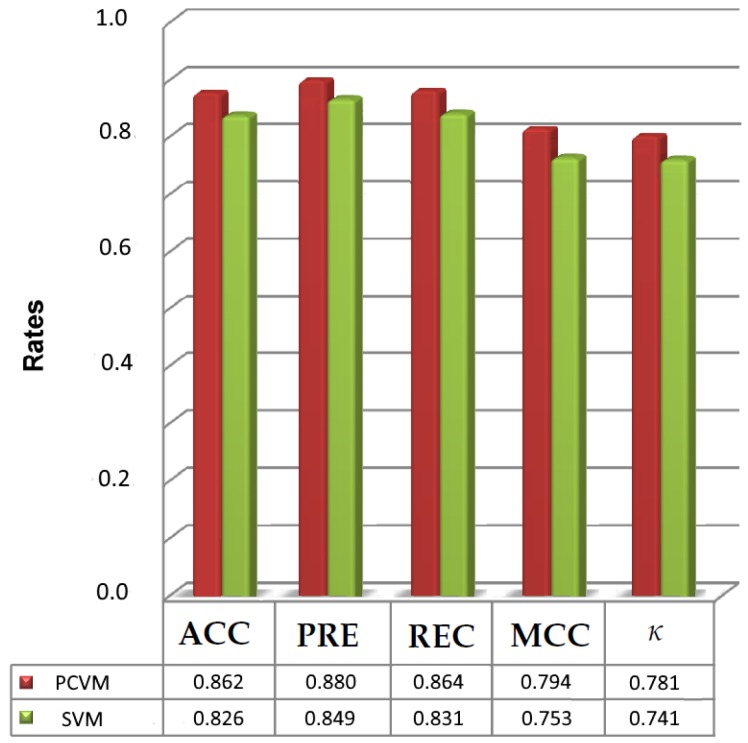
The maximum scores achieved for SVM and PCVM.

The analysis in Table 2, Table 3, Table 4, Table 5 and Table 6 show that the performance of PCVM has significantly outperformed SVM. Moreover, Figure 4 presents the maximum values recorded for PCVM and SVM. Both Table 2, Table 3, Table 4, Table 5 and Table 6 and Figure 4 reveal SHPCVM can be further used. Indeed, the performance of SVM is not so much competitive against the PCVM. The major reason PCVM is significantly better than SVM may be the fact that probabilistic decisions are important to accomplish such tasks.

#### 2.4.3. Comparison with Other Classification Methods

To further investigate the prediction performance of our approach, we also compared the proposed approach with several other existing methods on the GPCR datasets. The prediction results for the three additional classifiers and abovementioned measures are reported in Table 2, Table 3, Table 4, Table 5 and Table 6. One may observe that PCVM with a harmonic-based representation achieves the best results for all datasets. Table 2, Table 3, Table 4, Table 5 and Table 6 suggest the worst outcomes were provided by Naïve Bayes classifier. Some results are random in case of this approach. Take for instance the value for Q14416 or Q8TDU6 data presented in Table 5. It is probably caused by the fact that NB is a very a simple method that makes a strong assumption on the shape of the data distribution which may not be true for the analyzed datasets. Also, it can be seen in Table 2, Table 3, Table 4, Table 5 and Table 6 that RF and *K*NN results are poor. Generally, the outcomes show a common trend with the results for RF, *K*NN and NB, namely the results are much more worse than for either SVM or PCVM, but with specific differences due to the use of different classification methods.

#### 2.4.4. Comparison with Other Representations

**Figure 5:**
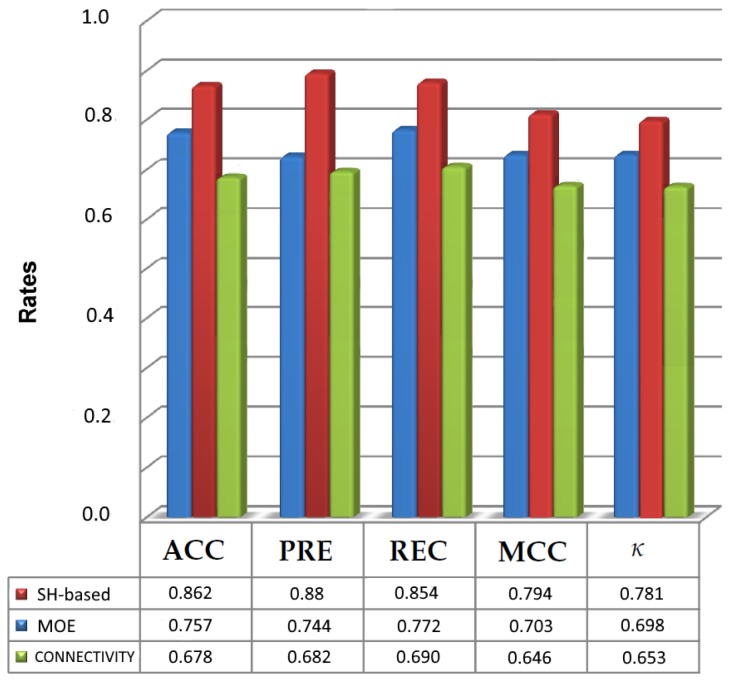
Maximum evaluation results obtained for the prediction of active molecules with spherical harmonic-based approach, MOE-type molecular descriptor and Connectivity descriptor using PCVM as the classifier.

To assess the ability of PCVM classifier, two existing descriptors, i.e., MOE (60 dimensions) and Connectivity (44 dimensions) found in RDKit [49], a popular cheminformatics package are applied to represent the GPCR datasets and the results are compared with the results of SH. The comparison of the results of these approaches in terms of Accuracy (ACC) and Matthews Correlation Coefficient (MCC) is listed in Table 7 and Table 8. Additionally, Figure 5 illustrates the maximum values obtained for each descriptor and PCVM when all measures are taken into consideration.

Table 7 suggests that the highest accuracy was obtained for SH-based variant and equals 0.862 in Q9Y5N1. Thus, from the results in Table 7 and Table 8, we can also conclude that the spherical harmonic-based representation was able to handle all the datasets. Most importantly, the results for harmonic-based representation (Table 7 and Table 8 and Figure 5) show that using SH-based as the descriptor has an influence on prediction of molecules activity. Although a harmonic-based representation has the same length as MOE-type descriptor, it has improved the effectiveness of the prediction of active molecules. The other results for the rest of datasets indicate that SHPCVM is very promising for molecular activity prediction and they are available in the Supplementary Materials.

## 3. Materials And Methods

In this section, we give a brief introduction to datasets we used for computer experiments. Then we introduce the details of PCVM, SVM, Random Forest, Bayesian classifier and *K*NN. Also, we present a brief introduction of representation descriptors, including characteristics of Spherical Harmonics-based approach.

### 3.1. Datasets

To get the data we partially repeated the steps described in [54]. We downloaded data for 3052 G-protein coupled receptors from UniProt database [55]. The database consists of 825 human GPCR proteins. Among these, we obtained 519 051 GPCR-ligand interactions data from the GLASS database [56]. For the purpose of ensuring the effectiveness of the computer experiments, we sorted the GPCRs by the number of interacting ligands, as done in [54]. Since some GPCR individuals have very small number of ligands or none, a threshold value to indicate the minimum number of ligands each target is expected to have is set to 600. Finally, we selected 21 proteins which are listed in Table 9. In consequence, there is a one individual which represents family F (Q99835), two representatives of class C (P41180, Q14416), one target from family B (P47871) and the additional representatives are associated with class A. All used ligands were gathered from CHEMBL database [57].

Several measures may be employed to verify the activity of molecules. They include $IC_{50}$, $EC_{50}$, $K_{i}$, $K_{d}$, etc. [58]. Thus, we followed the approach of Wu et al. [54] and the *p*-bioactivity is used in the work which is defined as $-\log_{10}val$. Please note that val is the raw bioactivity. The value of the raw bioactivities of ligands varies over a large range. However, taking logs reduces the magnitude of data in relation to other variables data, and the properties of the model were not lost in any case. In the datasets the activity range is extremely diverse. The smallest activity value is −12 and the largest is 4. For ligands which have more than one activity value, we assume the mean as the final *p*-bioactivity value. The inactive molecules are those which do not interact with the target GPCR. We selected them randomly from the set of irrelevant GPCR data, similarly as described in [54]. In consequence, the number of inactive compounds for a given GPCR target is about 30% of the actives (see Table 9). Unfortunately, the number of irrelevant datasets which are considered as inactive is smaller than the number of active compounds. This is the reason the data is unbalanced.

Please note that to solve the imbalanced data set problem, we have also made an attempt to select the compounds with the lowest activity data as inactive. In the experiments we have considered the values below −10. Taking such extra molecules decreased the results in the range of 0.222 to 0.375. We believe it was caused by the fact the low activity compounds were labeled as inactive.

### 3.2. Spherical Harmonics-Based Descriptor

To clearly introduce the Spherical Harmonics-based descriptor, we briefly introduce the concept of Spherical Harmonics and our feature selection idea in the following two subsections.

#### 3.2.1. Spherical Harmonics

Spherical harmonics are considered as a set of solutions to Laplace’s equation in spherical coordinates [80,81]. The coordinates construct a set of basis functions

(1) Ylm(θ,ϕ)=SlmPlm(cosθ)eImϕ,

where $P_{l}^{m}$ means the associated Legendre polynomials which are real-valued and defined over the range [-1,1]. The goal of $S_{l}^{m}$ is functions normalization.

(2) $$S_{l}^{m}\left(\theta ,\phi \right)=\sqrt[{}]{\frac{(2l+1)(l-m)\;!}{4\pi (l+m)\;!}}$$

We introduce the concept of spherical depth which is a function that provides the distance between two atoms. Thus, one can consider a molecule in a spherical depth map as a spherical function f(θ,ϕ) that may be expanded into a linear combination of all spherical harmonics scaled by their associated Fourier coefficients $c_{lm}$:

(3) $$f\left(\theta ,\phi \right)=\sum_{l=0}^{\infty }\sum_{m=-l}^{l}c_{l,m}Y_{l}^{m}\left(\theta ,\phi \right).$$

**Figure 6:**
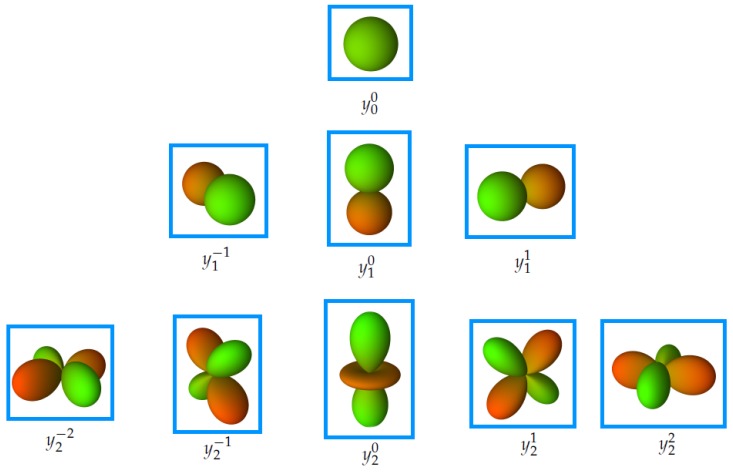
Illustration of the real valued spherical harmonic basis functions, where green means positive values and red is associated with negative values.

For molecular representation we need only real value spherical harmonics. The real valued spherical harmonic basis functions are shown in Figure 6. The real spherical harmonics can be expressed in spherical coordinates as follows:

(4) $$y_{l}^{m}\left(\theta ,\phi \right)=\left\{\begin{array}{c} \sqrt{2}S_{l}^{m}\cos\left(m\phi \right)P_{l}^{m}\cos\left(\theta \right);\;m>0\\ \sqrt{2}S_{l}^{m}\sin\left(-m\phi \right)P_{l}^{-m}\cos\left(\theta \right);\;m<0\\ S_{l}^{0}P_{l}^{0}\cos\left(\theta \right);\;m=0. \end{array}\right.$$

The spherical harmonic features (coefficients) are given by the equation:

(5) $$c_{l,m}=\int_{0}^{2\pi }\int_{0}^{\pi }f\left(\theta ,\phi \right)y_{l}^{m}\left(\theta ,\phi \right)\sin\left(\theta \right)d\theta d\phi$$

In consequence, the spherical harmonics descriptor is seen as a *k* dimensional vector

(6) $$V=(∥v_{1}∥,∥v_{2}∥,∥v_{3}∥,\cdots ,∥v_{d}∥),$$

where bandwidth that is important to achieve a certain concentration factor equals *N*, $∥v_{i}∥=\sqrt{\sum_{m=-l}^{l}{\left|c_{1,1}\right|}^{2}}$ and $d\left(V\right)\leq \frac{N}{2}$. Furthermore, *V* is rotation invariant.

#### 3.2.2. Feature Selection

Interestingly, it shows spherical harmonics are able to capture a various number of geometric object properties. The molecule’s model is characterized by the energies at different frequencies of spherical harmonics. Thus, at high frequencies one may capture some details, whereas low frequencies rather reveal gross information. In other words, for small value of *l* in Equation (5) we consider low frequencies and the higher value of *l* gives more details.

Nevertheless, the SH descriptor, itself, may produce numerous features. Obviously, it is one of many descriptors which may be employed to classification. However, the number of features included in the well-known descriptors (SH descriptor, among others) can be high. Such high dimensionality combined with a comparatively small sample size usually causes a degradation of the classifier’s performance. Such a phenomenon is known as the curse of dimensionality [82]. It shows a well-defined dimensionality reduction scheme may lead to an improvement in the performance of a prediction model. Feature selection algorithms reduce the dimensionality of the input sequence by selecting only a subset of features.

Feature selection approaches can be divided into filters [83] and wrappers [84]. Filters perform feature selection independently from the learning process. Wrappers combine the learning process and feature selection to select an optimal subset of features. Here, we apply Minimum Redundancy Maximum Relevance feature selection approach (MRMR) [85]. It represents a filter-based methodology. Generally, it selects highly predictive but uncorrelated features. The features are ranked according to the minimal-redundancy-maximal-relevance criteria.

Let us denote two random variables *X* and *Y*. Now, their mutual information is defined as:

(7) $$I\left(X,Y\right)=\int \int p\left(x,y\right)\log\frac{p(x,y)}{p(x)p(y)}dxdy,$$

where p(•) is the probability density function, *x* and *y* represent realizations *X* and *Y*. MRMR criterion is the following:

(8) maxψ(D,R),ψ=D-R,

where $max\;D\left(S,y\right)=\frac{1}{\left|S\right|}\sum_{x_{i}\in S}I\left(x_{i},y\right)$ (max relevance), $min\;R\left(S\right)=\frac{1}{{\left|S\right|}^{2}}\sum_{x_{i},x_{j}\in S}I\left(x_{i},x_{j}\right)$ (min redundancy) and *S* is the set of *n* input variables.

#### 3.2.3. Descriptor Computation

**Figure 7:**
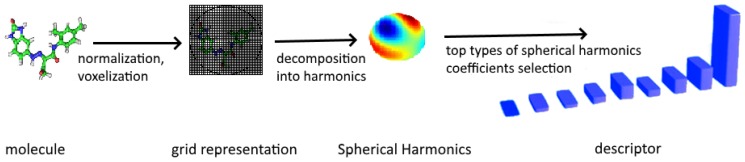
Steps in computing Spherical Harmonics-based descriptor.

To sum up, the procedure used to calculate our Spherical Harmonics-based descriptor includes the following steps which are also depicted in Figure 7.

1. Reading in atom’s type, coordinates, temperature factor, occupancy.
2. Placing a molecule into a common frame of reference.
3. Scaling in such a way each molecule fits within the unit ball.
4. Placing an orthogonal grid around each molecule.
5. Building so-called spherical depth map which provides the distance between the closest atoms.
6. Using the grid values to perform decomposition into spherical harmonics.
7. Learning the most informative Spherical Harmonics features by applying feature selection strategy Section 3.2.2 to the vector of coefficients given in (5) and (6).

In our approach, feature selection enables finding the most discriminative features (more precisely: type of features) before the training phase. Tests are performed on external data that was never used for neither feature selection nor training. All in all, SH-based descriptor is shorter than SH descriptor since it contains only the most descriptive types of features. Removing irrelevant features leads to the improvement in prediction and increases interpretability of the classification model.

Finally, the dimension of the descriptor presented in the paper is 60. The final length of 60 was chosen arbitrarily. We leave for further studies the challenges connected with the most optimal selection of number of coefficients. It is worth mentioning that since our SH-based descriptor depends on the 3D structure of the molecule, the molecular conformation has an influence on molecular prediction ability. In fact, it was out of the scope of this paper and we have not tested different conformations. Nevertheless, our studies suggest the more faithful 3D model is, the better Spherical Harmonic-based representation is expected to be. However, discussions on the impact of the 3D structure on SH-based representation could be a fruitful direction for future work.

### 3.3. Probabilistic Classification Vector Machines (PCVM)

Probabilistic Classification Vector Machines [86] is a probabilistic kernel classifier with a kernel regression model $\sum_{i}^{n}w_{i}\phi_{i,\theta }\left(x\right)+b$, where $w_{i}$ are the weights of the basis functions $\phi_{i,\theta }\left(x\right)$ and *b* is a bias. In the work we have adopted some PCVM settings to the molecules classification problem which is considered as a binary classification.

Suppose we have a dataset $S={\left\{x_{i},y_{i}\right\}}_{i=1}^{n}$, where $y_{i}\in \left\{-1,+1\right\}$ (labels - active and inactive molecules). We employed a probit link function

$$\psi \left(x\right)=\int_{-\infty }^{x}N\left(t|0,1\right)dt,$$

where ψ(x) is the cumulative distribution of the normal distribution. Expectation Maximization approach is used to optimize parameters. Finally, the model is defined as follows

$$l\left(x,w,b\right)=\psi \left(\sum_{i=1}^{n}w_{i,\phi }\left(x\right)+b\right)=\psi \left(\Phi_{\theta }\left(x\right)w+b\right),$$

where Ψ(x) is seen as a vector of basis function evaluations for a molecule *x*.

### 3.4. Other Approaches

Meanwhile, in order to further evaluate the performance of SHPCVM, we separately train the different state-of-the-art classifiers mentioned in the following subsections using Spherical Harmonics-based representation to encode the molecules.

#### 3.4.1. Support Vector Machines (SVM)

SVM [53] is a state-of-the art machine learning method that finds a hyperplane to separate data from different classes. SVM has been widely used in chemoinformatics and its generalization performance is significantly better than that of competing methods [87]. The choice of similarity measure is a vital step to increase the performance of SVM. Typically, a positive semi-definite similarity measure between data points (i.e., a kernel) is applied.

For the class of hyperplanes in a dot product space H, SVM performs a classification of samples using a decision function as follows:

f(x)=sgn(<w,x>+b),

where b∈R is the bias weight and w∈H are the feature weights.

For a linearly separable set of observations, a unique optimal hyperplane exists. It is differentiated by the maximal margin of separation between any observation point $x_{i}$ and the hyperplane. The optimal hyperplane is the solution of

$$\underset{b\in R,w\in H}{maximize}\min\{∥x-x_{i}∥;\;x\in H,\;<wx+b>=0,\;i=1,\cdots ,n\}.$$

In case of nonlinear decision function, the kernel trick is applied. *f* can be defined as:

$$f\left(x\right)=sgn(\sum_{i=1}^{n}y_{i}\alpha_{i}k\left(x,x_{i}\right)+b),$$

where k:H×H and $\left(x,x^{\prime }\right)\to k\left(x,x^{\prime }\right)$.

#### 3.4.2. Random Forests (RF)

A Random Forest is a supervised machine learning methodology that can be used to classify data into activity classes [88]. In formal, we consider a collection of randomized base regression trees $m_{n}\left(x,\Theta_{m},S_{n}\right)$, where $\Theta_{1},\Theta_{2},\cdots$ are associated with the randomness in the tree construction. Such random trees combined together form the aggregated regression estimate

$${\hat{m}}_{n}\left(X,S_{n}\right)=E_{\Theta }\left[m_{n}\left(X,\Theta ,S_{n}\right)\right],$$

where $S_{n}=\left\{\left(X_{1},Y_{1}\right),\left(X_{2},Y_{2}\right),\cdots ,\left(X_{n},Y_{n}\right)\right\}\subset R^{d}\times R$ is a training sample of independent and identically distributed random variables, E refers to the expectation with respect to the random parameter.

#### 3.4.3. *K* Nearest Neighbours (*K*NN)

*K* Nearest Neighbours classifier is a relatively simple classification model which uses a known dataset of molecules to classify a new compound by polling the closest data molecule in the known dataset. To be more precise, the new compound is classified based on the class with the majority representation among the *k* nearest neighbors.

The goal is to classify a new molecule mol∈M (made up of $m_{i}$, where i=1,⋯,|M|). Furthermore, each molecule $m_{i}$ is described by a set of features, ie. a vector $V_{i}=\left(f_{1},f_{2},f_{3},\cdots ,f_{n}\right)$ (descriptor). Formally, for each $m_{i}\in M$ the distance between a new molecule mol and $x_{i}$ is calculated.

$$\left(mol,x_{i}\right)=\sum_{f_{i}\in V_{i}}val_{f_{i}}\delta \left(mol_{f_{i}},m_{if_{i}}\right),$$

where δ(•) is a distance metric. Now, the voting strategy may be defined as follows

$$vote\_proc\left(y_{j}\right)=\sum_{c=1}^{k}\frac{1}{\left(mol,x_{c}\right)}\left(y_{j},y_{c}\right),$$

where $y_{j},y_{c}\in Y$ (set of labels - active and inactive).

#### 3.4.4. Naïve Bayes (NB)

Naïve Bayes classifier [89] is a linear classifier that assumes the features in a descriptor are mutually independent.

Suppose a given molecule *m* is assigned the activity class *a*

$$A^{*}=\arg\max_{a}\;p\left(a|m\right).$$

NB uses the Bayes’ rule

$$p\left(a|m\right)=\frac{p(a)p(m|a)}{p(m)}.$$

To estimate p(a|m), i.e., the probability of the molecule *m* being in class *a*, NB uses the following equation:

$$p_{NB}\left(a|m\right)=\frac{p\left(a\right)(\prod_{i=1}^{n}p{\left(V_{i}|a\right)}^{x_{i}\left(m\right)})}{p(m)},$$

where $V_{i}=\left(x_{1},x_{2},x_{3},\cdots ,x_{n}\right)$ is a feature vector that describes molecule *m*.

## 4. Conclusions

In this article, we propose a novel molecular activity prediction method called SHPCVM. More specifically, there are two main contributions of the paper.

- We have introduced the novel Spherical Harmonics-based descriptor. The key principle of our Spherical Harmonics-based approach is not the usage of Spherical Harmonics themselves but the fact that our technique makes use of feature selection strategy (Minimum Redundancy Maximum Relevance) that enables obtaining only representative features. We outline that such an approach leads to the development of a more interpretable representation. What is more important for us, the vector representation we get is relatively short and that affects the computational costs. Therefore, our approach has a significant impact on molecular activity prediction where one does not have a large set of labeled examples and low-dimensional descriptor is required.
- We have tested several machine learning methods, more precisely Probabilistic Classification Vector Machines (PCVM), Support Vector Machines (SVM), Naïve Bayes (NB) and *K* Nearest Neighbours (*K*NN) for molecules described by the proposed Spherical Harmonics-based model. The results yield Probabilistic Classification Vector Machines (PCVM) and Spherical Harmonics-based descriptor is superior to another approaches when molecular activity prediction of small compounds is considered. Obviously, the outcomes have revealed the influence of PCVM.

Experimental results for G protein-coupled receptors (GPCRs) demonstrate SHPCVM produces the best performance ranging from 0.742 Accuracy to 0.862, and from 0.691 to 0.794 in terms of Matthew Correlation Coefficient. Although the goal was to find out a tradeoff between the descriptive capabilities and computational costs of the descriptor, our approach may pave the way for more interpretability oriented research on molecule’s computational model.

## Figures and Tables

**Table 1:** Evaluation measures for the binary classification problem: TP—true positives (the total number of active compounds that are predicted correctly), TN—true negatives (the total number of inactive compounds that are predicted correctly), FP—false positives (the total number of these compounds that have no interaction with the receptor but are predicted as active), FN—false negatives (the total number of these compounds that are active but are predicted as inactive), $P_{A}$—an observed level of agreement, $P_{E}$—an expected level of agreement.

Measure	Computational Formula	Description
Accuracy [46]	$ACC=\frac{TP+TN}{TP+TN+FP+FN}$	It quantifies the fraction of correct predictions over the total instances.
Precision	$PRE=\frac{TP}{TP+FP}$	It quantifies the fraction of relevant instances among the retrieved ones.
Recall	$REC=\frac{TP}{TP+FN}$	It quantifies the fraction of relevant instances that have been retrieved over the total relevant instances.
Matthews Correlation Coefficient [47]	$MCC=\frac{TP\cdot TN-FP\cdot FN}{\sqrt{\left(TP+FP)(TP+FN)(TN+FP)(TN+FN\right)}}$	It returns a value between -1 and +1, where +1 represents a perfect prediction, -1 total disagreement between prediction and observation and 0 indicates no better than random prediction.
Cohen’s kappa [48]	$\kappa =\frac{P_{A}-P_{E}}{1-P_{E}}$	It returns a value between -1 and +1, where +1 represents a complete agreement, 0 or lower values mean chance agreement.

**Table 2:** Performance comparison of target prediction methods in terms of Accuracy. Scores for the external test set.

UniProt ID	PCVM	SVM	RF	NB	*K*NN
P35372	0.820	0.771	0.694	0.636	0.659
P30542	0.742	0.712	0.637	0.608	0.595
P08908	0.809	0.750	0.671	0.603	0.632
Q9Y5N1	0.862	0.826	0.745	0.676	0.703
Q99705	0.814	0.788	0.716	0.659	0.694
Q14416	0.804	0.752	0.672	0.585	0.657
P21917	0.776	0.721	0.644	0.573	0.608
Q9HC97	0.770	0.741	0.658	0.596	0.621
Q99835	0.854	0.812	0.736	0.664	0.682
P50406	0.821	0.794	0.704	0.598	0.639
Q8TDU6	0.830	0.802	0.732	0.672	0.699
P47871	0.831	0.762	0.697	0.648	0.646
P30968	0.801	0.774	0.666	0.589	0.634
P35348	0.821	0.789	0.761	0.678	0.747
P24530	0.830	0.802	0.734	0.687	0.717
P41180	0.842	0.816	0.723	0.659	0.664
P51677	0.800	0.814	0.667	0.596	0.633
P21452	0.805	0.809	0.683	0.632	0.631
P35346	0.772	0.742	0.699	0.618	0.629
P48039	0.799	0.760	0.696	0.629	0.658
Q9Y5Y4	0.821	0.773	0.701	0.623	0.659

**Table 3:** Performance comparison of target prediction methods in terms of Precision. Scores for the external test set.

UniProt ID	PCVM	SVM	RF	NB	*K*NN
P35372	0.807	0.761	0.663	0.629	0.584
P30542	0.726	0.696	0.619	0.547	0.501
P08908	0.808	0.763	0.675	0.633	0.613
Q9Y5N1	0.889	0.849	0.723	0.644	0.674
Q99705	0.832	0.814	0.708	0.657	0.675
Q14416	0.791	0.772	0.609	0.566	0.575
P21917	0.732	0.681	0.618	0.547	0.581
Q9HC97	0.761	0.738	0.673	0.533	0.649
Q99835	0.867	0.830	0.718	0.642	0.692
P50406	0.827	0.791	0.691	0.615	0.653
Q8TDU6	0.821	0.794	0.673	0.597	0.622
P47871	0.822	0.765	0.693	0.612	0.634
P30968	0.790	0.762	0.638	0.621	0.629
P35348	0.812	0.777	0.686	0.639	0.648
P24530	0.815	0.783	0.707	0.648	0.643
P41180	0.863	0.834	0.712	0.615	0.638
P51677	0.803	0.818	0.688	0.595	0.657
P21452	0.791	0.791	0.643	0.534	0.629
P35346	0.804	0.777	0.677	0.592	0.652
P48039	0.786	0.752	0.642	0.569	0.639
Q9Y5Y4	0.816	0.760	0.716	0.628	0.656

**Table 4:** Performance comparison of target prediction methods in terms of Recall. Scores for the external test set.

UniProt ID	PCVM	SVM	RF	NB	*K*NN
P35372	0.826	0.783	0.668	0.626	0.596
P30542	0.752	0.725	0.651	0.533	0.456
P08908	0.786	0.738	0.677	0.669	0.585
Q9Y5N1	0.847	0.816	0.675	0.655	0.676
Q99705	0.798	0.775	0.686	0.639	0.623
Q14416	0.808	0.819	0.602	0.542	0.569
P21917	0.764	0.713	0.621	0.536	0.597
Q9HC97	0.787	0.757	0.671	0.522	0.616
Q99835	0.826	0.797	0.689	0.616	0.634
P50406	0.788	0.764	0.687	0.598	0.631
Q8TDU6	0.841	0.819	0.676	0.552	0.593
P47871	0.854	0.801	0.688	0.578	0.648
P30968	0.835	0.803	0.651	0.655	0.623
P35348	0.853	0.817	0.675	0.602	0.619
P24530	0.864	0.831	0.664	0.626	0.607
P41180	0.824	0.793	0.693	0.619	0.609
P51677	0.822	0.795	0.683	0.513	0.615
P21452	0.820	0.781	0.634	0.506	0.595
P35346	0.764	0.739	0.686	0.569	0.615
P48039	0.814	0.784	0.649	0.593	0.625
Q9Y5Y4	0.840	0.791	0.676	0.625	0.646

**Table 5:** Performance comparison of target prediction methods in terms of Matthews Correlation Coefficient. Scores for the external test set.

UniProt ID	PCVM	SVM	RF	NB	*K*NN
P35372	0.768	0.725	0.611	0.573	0.557
P30542	0.691	0.654	0.648	0.552	0.387
P08908	0.756	0.702	0.652	0.606	0.544
Q9Y5N1	0.765	0.738	0.635	0.588	0.614
Q99705	0.770	0.746	0.632	0.577	0.593
Q14416	0.714	0.715	0.577	0.504	0.514
P21917	0.783	0.733	0.619	0.465	0.552
Q9HC97	0.696	0.661	0.633	0.480	0.603
Q99835	0.751	0.729	0.656	0.613	0.615
P50406	0.777	0.748	0.664	0.556	0.611
Q8TDU6	0.773	0.746	0.637	0.511	0.582
P47871	0.794	0.748	0.656	0.557	0.615
P30968	0.774	0.741	0.606	0.614	0.577
P35348	0.764	0.727	0.637	0.609	0.595
P24530	0.787	0.751	0.625	0.572	0.596
P41180	0.781	0.753	0.655	0.596	0.563
P51677	0.753	0.724	0.627	0.485	0.618
P21452	0.766	0.721	0.569	0.473	0.588
P35346	0.690	0.664	0.638	0.566	0.603
P48039	0.742	0.717	0.617	0.593	0.582
Q9Y5Y4	0.754	0.701	0.625	0.625	0.595

**Table 6:** Performance comparison of target prediction methods in terms of κ. Scores for the external test set.

UniProt ID	PCVM	SVM	RF	NB	*K*NN
P35372	0.727	0.682	0.617	0.548	0.551
P30542	0.651	0.615	0.624	0.552	0.377
P08908	0.740	0.697	0.622	0.623	0.544
Q9Y5N1	0.742	0.684	0.611	0.566	0.612
Q99705	0.751	0.698	0.624	0.557	0.622
Q14416	0.689	0.676	0.534	0.472	0.556
P21917	0.772	0.722	0.621	0.467	0.565
Q9HC97	0.663	0.634	0.613	0.474	0.587
Q99835	0.732	0.703	0.648	0.635	0.573
P50406	0.761	0.734	0.622	0.519	0.588
Q8TDU6	0.766	0.731	0.623	0.512	0.542
P47871	0.781	0.735	0.636	0.559	0.622
P30968	0.763	0.732	0.595	0.613	0.564
P35348	0.750	0.725	0.654	0.544	0.575
P24530	0.753	0.722	0.603	0.568	0.591
P41180	0.772	0.741	0.625	0.587	0.543
P51677	0.735	0.691	0.586	0.467	0.582
P21452	0.723	0.687	0.528	0.456	0.557
P35346	0.668	0.633	0.608	0.547	0.579
P48039	0.713	0.680	0.575	0.557	0.564
Q9Y5Y4	0.742	0.691	0.592	0.617	0.592

**Table 7:** Performance comparison of target prediction methods in terms of Accuracy. Scores for the external test set.

UniProt ID	SH-Based					MOE-Type					Connectivity				
	PCVM	SVM	RF	NB	*K*NN	PCVM	SVM	RF	NB	*K*NN	PCVM	SVM	RF	NB	*K*NN
P35372	0.820	0.771	0.694	0.636	0.659	0.734	0.725	0.651	0.604	0.587	0.669	0.685	0.616	0.562	0.551
P30542	0.742	0.712	0.637	0.608	0.595	0.691	0.708	0.617	0.615	0.623	0.633	0.653	0.604	580	0.566
P08908	0.809	0.750	0.671	0.603	0.632	0.731	0.746	0.673	0.643	0.604	0.606	0.648	0.583	541	0.569
Q9Y5N1	0.862	0.826	0.745	0.676	0.703	0.713	0.708	0.662	0.629	0.591	0.607	0.622	0.571	0.553	0.512
Q99705	0.814	0.788	0.716	0.659	0.694	0.731	0.713	0.685	0.641	0.611	0.678	0.721	0.645	0.609	0.621
Q14416	0.804	0.752	0.672	0.585	0.657	0.712	0.695	0.651	0.612	0.576	0.649	0.628	0.604	0.584	0.568
P21917	0.776	0.721	0.644	0.573	0.608	0.722	0.672	0.616	0.583	0.562	0.641	0.627	0.598	0.567	0.557
Q9HC97	0.770	0.741	0.658	0.596	0.621	0.664	0.673	0.607	0.617	0.573	0.602	0.616	0.573	0.552	0.564
Q99835	0.854	0.812	0.736	0.664	0.682	0.732	0.716	0.668	0.613	0.563	0.669	0.653	0.606	0.581	0.566
P50406	0.821	0.794	0.704	0.598	0.639	0.695	0.711	0.672	0.605	0.568	0.592	0.575	0.542	0.527	0.511
Q8TDU6	0.830	0.802	0.732	0.672	0.699	0.616	0.654	0.632	0.616	0.584	0.511	0.561	0.548	0.539	0.525
P47871	0.831	0.762	0.697	0.648	0.646	0.757	0.718	0.672	0.649	0.622	0.610	0.628	0.572	0.548	0.525
P30968	0.801	0.774	0.666	0.589	0.634	0.712	0.697	0.685	0.574	0.592	0.622	0.641	0.579	0.526	0.503
P35348	0.821	0.789	0.761	0.678	0.747	0.728	0.735	0.678	0.638	0.603	0.593	0.604	0.561	0.539	0.558
P24530	0.830	0.802	0.734	0.687	0.717	0.712	0.759	0.663	0.625	0.611	0.584	0.616	0.559	0.539	0.593
P41180	0.842	0.816	0.723	0.659	0.664	0.716	0.736	0.671	0.614	0.592	0.608	0.585	0.553	0.528	0.542
P51677	0.800	0.814	0.667	0.596	0.633	0.625	0.672	0.633	0.582	0.606	0.559	0.586	0.531	0.502	0.484
P21452	0.805	0.809	0.683	0.632	0.631	0.641	0.639	0.625	0.593	0.613	0.534	0.556	0.502	0.528	0.502
P35346	0.772	0.742	0.699	0.618	0.629	0.658	0.692	0.685	0.572	0.589	0.542	0.568	0.511	0.518	0.528
P48039	0.799	0.760	0.696	0.629	0.658	0.692	0.713	0.652	0.585	0.603	0.590	0.623	0.584	0.548	0.523
Q9Y5Y4	0.821	0.773	0.701	0.623	0.659	0.739	0.758	0.649	0.578	0.559	0.630	0.641	0.596	0.542	0.531

**Table 8:** Performance comparison of target prediction methods in terms of Matthews Correlation Coefficient. Scores for the external test set.

UniProt ID	SH-Based					MOE-Type					Connectivity				
	PCVM	SVM	RF	NB	*K*NN	PCVM	SVM	RF	NB	*K*NN	PCVM	SVM	RF	NB	*K*NN
P35372	0.768	0.725	0.611	0.573	0.557	0.654	0.623	0.599	0.551	0.506	0.646	0.618	0.588	0.539	0.526
P30542	0.691	0.654	0.648	0.552	0.387	0.588	0.553	0.507	0.523	0.501	0.528	0.514	0.485	0.503	0.495
P08908	0.756	0.702	0.652	0.606	0.544	0.702	0.664	0.615	0.602	0.610	0.402	0.443	0.482	0.506	0.501
Q9Y5N1	0.765	0.738	0.635	0.588	0.614	0.601	0.572	0.548	0.563	0.556	0.488	0.509	0.512	0.519	0.489
Q99705	0.770	0.746	0.632	0.577	0.593	0.637	0.612	0.585	0.511	0.594	0.587	0.575	0.559	0.508	0.569
Q14416	0.714	0.715	0.577	0.504	0.514	0.613	0.624	0.619	0.572	0.603	0.584	0.602	0.564	0.581	0.568
P21917	0.783	0.733	0.619	0.465	0.552	0.671	0.693	0.642	0.618	0.637	0.560	0.613	0.582	0.554	0.549
Q9HC97	0.696	0.661	0.633	0.480	0.603	0.586	0.591	0.544	0.531	0.505	0.537	0.558	0.521	0.506	0.502
Q99835	0.751	0.729	0.656	0.613	0.615	0.684	0.702	0.638	0.597	0.582	0.632	0.613	0.574	0.607	0.601
P50406	0.777	0.748	0.664	0.556	0.611	0.648	0.668	0.624	0.539	0.556	0.481	0.446	0.503	0.501	0.495
Q8TDU6	0.773	0.746	0.637	0.511	0.582	0.529	0.516	0.495	0.502	0.505	0.421	0.376	0.504	0.508	0.481
P47871	0.794	0.748	0.656	0.557	0.615	0.635	0.659	0.622	0.575	0.599	0.531	0.578	0.593	0.504	0.512
P30968	0.774	0.741	0.606	0.614	0.577	0.583	0.603	0.558	0.506	0.540	0.522	0.536	0.495	0.552	0.506
P35348	0.764	0.727	0.637	0.609	0.595	0.632	0.667	0.613	0.582	0.571	0.531	0.554	0.496	0.517	0.554
P24530	0.787	0.751	0.625	0.572	0.596	0.641	0.685	0.597	0.562	0.610	0.530	0.579	0.516	0.503	0.526
P41180	0.781	0.753	0.655	0.596	0.563	0.687	0.641	0.582	0.545	0.569	0.531	0.552	0.506	0.491	0.507
P51677	0.753	0.724	0.627	0.485	0.618	0.602	0.628	0.564	0.550	0.586	0.489	0.439	0.501	0.518	0.493
P21452	0.766	0.721	0.569	0.473	0.588	0.618	0.616	0.582	0.547	0.593	0.473	0.491	0.464	0.414	0.402
P35346	0.690	0.664	0.638	0.566	0.603	0.564	0.575	0.532	0.516	0.551	0.481	0.452	0.471	0.418	0.459
P48039	0.742	0.717	0.617	0.593	0.582	0.609	0.658	0.604	0.613	0.585	0.489	0.496	0.452	0.549	0.512
Q9Y5Y4	0.754	0.701	0.625	0.625	0.595	0.703	0.684	0.642	0.605	0.668	0.582	0.573	0.551	0.512	0.560

**Table 9:** Datasets used in the experiments.

UniProt ID	Protein Name	# of Actives	# of Inactives
P35372	Mu-type opioid receptor [59]	3828	1100
P30542	Adenosine receptor A1 [60]	3016	900
P08908	5-Hydroxytryptamine receptor 1A [61]	2294	700
Q9Y5N1	Histamine H3 receptor [62]	2092	600
Q99705	Melanin-concentrating hormone receptors 1 [63]	2052	600
Q14416	Metabotropic glutamate receptor 2 [64]	1810	540
P21917	D(4) dopamine receptor [65]	1679	500
Q9HC97	G-protein coupled receptor 35 [66]	1589	470
Q99835	Smoothened homolog [67]	1523	450
P50406	5-Hydroxytryptamine receptor 6 [68]	1421	420
Q8TDU6	G-protein coupled bile acid receptor 1 [69]	1153	340
P47871	Glucagon receptor [70]	1129	340
P30968	Gonadotropin-releasing hormone receptor [71]	1124	340
P35348	Alpha-1A adrenergic receptor [72]	1027	300
P24530	Endothelin receptor type B [73]	1019	305
P41180	Extracellular calcium-sensing receptor [74]	940	280
P51677	C-C chemokine receptor type 3 [75]	781	234
P21452	Substance-K receptor [76]	696	170
P35346	Somatostatin receptor type 5 [77]	689	200
P48039	Melatonin receptor type 1A [78]	684	200
Q9Y5Y4	Prostaglandin D2 receptor 2 [79]	641	190

## Supplementary Materials

Supplementary materials can be found at https://www.mdpi.com/1422-0067/20/9/2175/s1.

## References

[1] Jazayeri A., Dias J., Marshall F. (2015). From G Protein-coupled Receptor Structure Resolution to Rational Drug Design. J. Biol. Chem..

[2] Ramsay R.R., Popovic-Nikolic M.R., Nikolic K., Uliassi E., Bolognesi M.L. (2018). A perspective on multi-target drug discovery and design for complex diseases. Clin. Transl. Med..

[3] Reddy A.S., Zhang S. (2013). Polypharmacology: Drug discovery for the future. Expert Rev. Clin. Pharmacol..

[4] Rester U. (2008). From virtuality to reality—Virtual screening in lead discovery and lead optimization: A medicinal chemistry perspective. Curr. Opin. Drug Discov. Dev..

[5] Srinivas R., Klimovich P.V., Larson E.C. (2018). Implicit-descriptor ligand-based virtual screening by means of collaborative filtering. J. Cheminform..

[6] Willett P., Wilton D.J., Hartzoulakis B., Tang R., Ford J., Madge D. (2007). Prediction of Ion Channel Activity Using Binary Kernel Discrimination. J. Chem. Inf. Model..

[7] Smusz S., Kurczab R., Bojarski A. (2013). A multidimensional analysis of machine learning methods performance in the classification of bioactive compounds. Chemom. Intell. Lab. Syst..

[8] Nidhi, Glick M., Davies J.W., Jenkins J.L. (2006). Prediction of Biological Targets for Compounds Using Multiple-Category Bayesian Models Trained on Chemogenomics Databases. J. Chem. Inf. Model..

[9] Xia X., Maliski E.G., Gallant P., Rogers D. (2004). Classification of Kinase Inhibitors Using a Bayesian Model. J. Med. Chem..

[10] Buchwald F., Richter L., Kramer S. (2011). Predicting a small molecule-kinase interaction map: A machine learning approach. J. Cheminform..

[11] Bruce C.L., Melville J.L., Pickett S.D., Hirst J.D. (2007). Contemporary QSAR Classifiers Compared. J. Chem. Inf. Model..

[12] Czarnecki W.M., Podlewska S., Bojarski A.J. (2015). Robust optimization of SVM hyperparameters in the classification of bioactive compounds. J. Cheminform..

[13] Rataj K., Czarnecki W., Podlewska S., Pocha A., Bojarski A.J. (2018). Substructural Connectivity Fingerprint and Extreme Entropy Machines—A New Method of Compound Representation and Analysis. Molecules.

[14] Zhang S., Hao L.Y., Zhang T.H. (2014). Prediction of Protein–Protein Interaction with Pairwise Kernel Support Vector Machine. Int. J. Mol. Sci..

[15] Liu B., Wang S., Dong Q., Li S., Liu X. (2016). Identification of DNA-Binding Proteins by Combining Auto-Cross Covariance Transformation and Ensemble Learning. IEEE Trans. Nanobiosci..

[16] Todeschini R., Consonni V. (2009). Molecular Descriptors for Chemoinformatics.

[17] Bartók A.P., Kondor R., Csányi G. (2013). On representing chemical environments. Phys. Rev. B.

[18] Lo Y.C., Rensi S.E., Torng W., Altman R.B. (2018). Machine learning in chemoinformatics and drug discovery. Drug Discov. Today.

[19] Hansch C., Muir R.M., Fujita T., Maloney P.P., Geiger F., Streich M. (1963). The Correlation of Biological Activity of Plant Growth Regulators and Chloromycetin Derivatives with Hammett Constants and Partition Coefficients. J. Am. Chem. Soc..

[20] Neves B.J., Braga R.C., Melo-Filho C.C., Moreira-Filho J.T., Muratov E.N., Andrade C.H. (2018). QSAR-Based Virtual Screening: Advances and Applications in Drug Discovery. Front. Pharmacol..

[21] Cherkasov A., Muratov E.N., Fourches D., Varnek A., Baskin I.I., Cronin M., Dearden J., Gramatica P., Martin Y.C., Todeschini R. (2014). QSAR Modeling: Where Have You Been? Where Are You Going to?. J. Med. Chem..

[22] Tropsha A. (2010). Best Practices for QSAR Model Development, Validation, and Exploitation. Mol. Inform..

[23] Kausar S., Falcao A.O. (2018). An automated framework for QSAR model building. J. Cheminform..

[24] Lozano N.B.H., de Oliveira R.F., Weber K.C., Honorio K.M., Guido R.V.C., Andricopulo A.D., da Silva A.B.F. (2013). Identification of Electronic and Structural Descriptors of Adenosine Analogues Related to Inhibition of Leishmanial Glyceraldehyde-3-Phosphate Dehydrogenase. Molecules.

[25] Adeniji S.E., Uba S., Uzairu A. (2018). QSAR Modeling and Molecular Docking Analysis of Some Active Compounds against Mycobacterium tuberculosis Receptor (Mtb CYP121). J. Pathog..

[26] Barley M.H., Turner N.J., Goodacre R. (2018). Improved Descriptors for the Quantitative Structure–Activity Relationship Modeling of Peptides and Proteins. J. Chem. Inf. Model..

[27] Tong W., Hong H., Xie Q., Shi L., Fang H., Perkins R. (2005). Assessing QSAR limitations—A regulatory perspective. Curr. Comput. Aided Drug Des..

[28] Ghasemi F., Mehridehnavi A., Pérez-Garrido A., Pérez-Sánchez H. (2018). Neural network and deep-learning algorithms used in QSAR studies: Merits and drawbacks. Drug Discov. Today.

[29] Consonni V., Todeschini R., Ballabio D., Grisoni F. (2019). On the Misleading Use of for QSAR Model Comparison. Mol. Inform..

[30] Bengio Y., Courville A., Vincent P. (2013). Representation Learning: A Review and New Perspectives. IEEE Trans. Pattern Anal. Mach. Intell..

[31] Kuroda M. (2017). A novel descriptor based on atom-pair properties. J. Cheminform..

[32] Śmieja M., Warszycki D. (2016). Average Information Content Maximization—A New Approach for Fingerprint Hybridization and Reduction. PLoS ONE.

[33] Winter R., Montanari F., Noé F., Clevert D.A. (2019). Learning continuous and data-driven molecular descriptors by translating equivalent chemical representations. Chem. Sci..

[34] Wang Y., You Z., Li X., Chen X., Jiang T., Zhang J. (2017). PCVMZM: Using the Probabilistic Classification Vector Machines Model Combined with a Zernike Moments Descriptor to Predict Protein–Protein Interactions from Protein Sequences. Int. J. Mol. Sci..

[35] Li L.P., Wang Y.B., You Z.H., Li Y., An J.Y. (2018). PCLPred: A Bioinformatics Method for Predicting Protein–Protein Interactions by Combining Relevance Vector Machine Model with Low-Rank Matrix Approximation. Int. J. Mol. Sci..

[36] Wang J., Zhang L., Jia L., Ren Y., Yu G. (2017). Protein-Protein Interactions Prediction Using a Novel Local Conjoint Triad Descriptor of Amino Acid Sequences. Int. J. Mol. Sci..

[37] Yuan X., Xu Y. (2018). Recent Trends and Applications of Molecular Modeling in GPCR–Ligand Recognition and Structure-Based Drug Design. Int. J. Mol. Sci..

[38] Jastrzębski S., Sieradzki I., Leśniak D., Tabor J., Bojarski A.J., Podlewska S. (2018). Three-dimensional descriptors for aminergic GPCRs: Dependence on docking conformation and crystal structure. Mol. Divers..

[39] Basith S., Cui M., Macalino S.J.Y., Park J., Clavio N.A.B., Kang S., Choi S. (2018). Exploring G Protein-Coupled Receptors (GPCRs) Ligand Space via Cheminformatics Approaches: Impact on Rational Drug Design. Front. Pharmacol..

[40] Sriram K., Insel P.A. (2018). GPCRs as targets for approved drugs: How many targets and how many drugs?. Mol. Pharmacol..

[41] Wang Q., Birod K., Angioni C., Grösch S., Geppert T., Schneider P., Rupp M., Schneider G. (2011). Spherical Harmonics Coefficients for Ligand-Based Virtual Screening of Cyclooxygenase Inhibitors. PLoS ONE.

[42] Ding L., Levesque M., Borgis D., Belloni L. (2017). Efficient molecular density functional theory using generalized spherical harmonics expansions. J. Chem. Phys..

[43] Bai L.Y., Dai H., Xu Q., Junaid M., Peng S.L., Zhu X., Xiong Y., Wei D.Q. (2018). Prediction of Effective Drug Combinations by an Improved Naïve Bayesian Algorithm. Int. J. Mol. Sci..

[44] Radovic M., Ghalwash M., Filipovic N., Obradovic Z. (2017). Minimum redundancy maximum relevance feature selection approach for temporal gene expression data. BMC Bioinform..

[45] Qiao Y., Xiong Y., Gao H., Zhu X., Chen P. (2018). Protein-protein interface hot spots prediction based on a hybrid feature selection strategy. BMC Bioinform..

[46] Gu Q., Zhu L., Cai Z., Cai Z., Li Z., Kang Z., Liu Y. (2009). Evaluation Measures of the Classification Performance of Imbalanced Data Sets. Computational Intelligence and Intelligent Systems.

[47] Matthews B.W. (1975). Comparison of the predicted and observed secondary structure of T4 phage lysozyme. Biochim. Biophys. Acta.

[48] Cohen J. (1960). A Coefficient of Agreement for Nominal Scales. Educ. Psychol. Meas..

[49] Landrum G. RDKit: Open-Source Cheminformatics. http://www.rdkit.org.

[50] O’Boyle N.M., Banck M., James C.A., Morley C., Vandermeersch T., Hutchison G.R. (2011). Open Babel: An open chemical toolbox. J. Cheminform..

[51] Cao D.S., Hu Q.N., Xu Q.S., Liang Y.Z. (2013). ChemoPy: Freely available python package for computational biology and chemoinformatics. Bioinformatics.

[52] Jolliffe I. (1986). Principal Component Analysis.

[53] Cortes C., Vapnik V. (1995). Support-Vector Networks. Mach. Learn..

[54] Wu J., Zhang Y., Hu H., Zhang Q., Wu W., Pang T., Chan W.K.B., Ke X. (2018). WDL-RF: Predicting bioactivities of ligand molecules acting with G protein-coupled receptors by combining weighted deep learning and random forest. Bioinformatics.

[55] UniProt Consortium T. (2018). UniProt: The universal protein knowledgebase. Nucleic Acids Res..

[56] Özgür A., Zhang H., Brender J.R., Yang J., Hur J., Chan W.K.B., Zhang Y. (2015). GLASS: A comprehensive database for experimentally validated GPCR-ligand associations. Bioinformatics.

[57] Gaulton A., Bellis L.J., Bento A.P., Chambers J., Davies M., Hersey A., Light Y., McGlinchey S., Michalovich D., Al-Lazikani B. (2012). ChEMBL: A large-scale bioactivity database for drug discovery. Nucleic Acids Res..

[58] Cortes-Ciriano I. (2016). Benchmarking the Predictive Power of Ligand Efficiency Indices in QSAR. J. Chem. Inf. Model..

[59] Liu X., Liu Z.C., Sun Y.G., Ross M., Kim S., Tsai F.F., Li Q.F., Jeffry J., Kim J.Y., H Loh H., Chen Z.F. (2011). Unidirectional Cross-activation of GRPR by MOR1D Uncouples Itch and Analgesia Induced by Opioids. Cell.

[60] Phillis J. (2004). Adenosine and Adenine Nucleotides as Regulators of Cerebral Blood Flow: Roles of Acidosis, Cell Swelling, and KATP Channels. Crit. Rev. Neurobiol..

[61] Ito H., Halldin C., Farde L. (1999). Localization of 5-HT1A receptors in the living human brain using [carbonyl-11C]WAY-100635: PET with anatomic standardization technique. J. Nucl. Med. Off. Publ. Soc. Nucl. Med..

[62] Esbenshade T.A., Browman K.E., Bitner R.S., Strakhova M.I., Cowart M.D., Brioni J.D. (2008). The histamine H3 receptor: An attractive target for the treatment of cognitive disorders. Br. J. Pharmacol..

[63] Rivera G., Bocanegra-Garcia V., Galiano S., Cirauqui Diaz N., Ceras J., Pérez S., Aldana I., Monge A. (2008). Melanin-Concentrating Hormone Receptor 1 Antagonists: A New Perspective for the Pharmacologic Treatment of Obesity. Curr. Med. Chem..

[64] Flor P.J., Lindauer K., Püttner I., Rüegg D., Lukic S., Knöpfel T., Kuhn R. (1995). Molecular Cloning, Functional Expression and Pharmacological Characterization of the Human Metabotropic Glutamate Receptor Type 2. Eur. J. Neurosci..

[65] Zhang J., Yang J., Jang R., Zhang Y. (2015). GPCR-I-TASSER: A Hybrid Approach to G Protein-Coupled Receptor Structure Modeling and the Application to the Human Genome. Structure.

[66] Shrimpton A., Braddock B., Thomson L., Stein C., Hoo J. (2004). Molecular delineation of deletions on 2q37.3 in three cases with an Albright hereditary osteodystrophy-like phenotype. Clin. Genet..

[67] van den Heuvel M., Ingham P. (1996). Smoothened encodes a receptor-like serpentine protein required for hedgehog signalling. Nature.

[68] Woolley M.L., Marsden C.A., Fone K.C.F. (2004). 5-ht6 receptors. Curr. Drug Targets. CNS Neurol. Disord..

[69] Wang Y., Chen W., Yu D.D., Forman B.M., Huang W. (2011). The G-protein-coupled bile acid receptor, Gpbar1 (TGR5), negatively regulates hepatic inflammatory response through antagonizing nuclear factor *κ* light-chain enhancer of activated B cells (NF-*κ*B) in mice. Hepatology.

[70] Hager J., Hansen L., Vaisse C., Vionnet N., Philippi A., Poller W., Velho G., Carcassi C., Contu L., Julier C. (1995). A Missense Mutation in the Glucagon Receptor Gene is Associated with Non-insulin-dependent Diabetes Mellitus. Nat. Genet..

[71] Chan Y.M., de Guillebon A., Lang-Muritano M., Plummer L., Cerrato F., Tsiaras S., Gaspert A., Lavoie H.B., Wu C.H., Crowley W.F. (2009). GNRH1 mutations in patients with idiopathic hypogonadotropic hypogonadism. Proc. Natl. Acad. Sci. USA.

[72] Thomas R.C., Cowley P.M., Singh A., Myagmar B.E., Swigart P.M., Baker A.J., Simpson P.C. (2016). The Alpha-1A Adrenergic Receptor in the Rabbit Heart. PLoS ONE.

[73] Tanaka H., Moroi K., Iwai J., Takahashi H., Ohnuma N., Hori S., Takimoto M., Nishiyama M., Masaki T., Yanagisawa M. (1998). Novel Mutations of the Endothelin B Receptor Gene in Patients with Hirschsprung’s Disease and Their Characterization. J. Biol. Chem..

[74] Kim J.Y., Ho H., Kim N., Liu J., Tu C.L., Yenari M.A., Chang W. (2014). Calcium-sensing receptor (CaSR) as a novel target for ischemic neuroprotection. Ann. Clin. Transl. Neurol..

[75] Choe H., Farzan M., Sun Y., Sullivan N., Rollins B., Ponath P.D., Wu L., Mackay C.R., LaRosa G., Newman W. (1996). The beta-chemokine receptors CCR3 and CCR5 facilitate infection by primary HIV-1 isolates. Cell.

[76] Baichwal V.R., Hammerschmidt W., Sugden B., Knippers R., Levine A.J. (1989). Characterization of the BNLF-1 Oncogene of Epstein-Barr Virus. Transforming Proteins of DNA Tumor Viruses.

[77] Tulipano G., Bonfanti C., Milani G., Billeci B., Bollati A., Cozzi R., Maira G., Murphy W.J., Poiesi C., Turazzi S. (2001). Differential inhibition of growth hormone secretion by analogs selective for somatostatin receptor subtypes 2 and 5 in human growth-hormone-secreting adenoma cells in vitro. Neuroendocrinology.

[78] Slaugenhaupt S.A., Roca A., Liebert C.B., Altherr M.R., Gusella J.F., Reppert S.M. (1995). Mapping of the Gene for the Mel1a-Melatonin Receptor to Human Chromosome 4 (MTNR1A) and Mouse Chromosome 8 (Mtnr1a). Genomics.

[79] Nantel F., Fong C., Lamontagne S., Hamish Wright D., Giaid A., Desrosiers M., Metters K.M., O’Neill G.P., Gervais F. (2004). Expression of prostaglandin D synthase and the prostaglandin D2 receptors DP and CRTH2 in human nasal mucosa. Prostaglandins Other Lipid Mediat..

[80] Vranic D.V., Saupe D., Richter J. Tools for 3D-object retrieval: Karhunen-Loeve transform and spherical harmonics. Proceedings of the 2001 IEEE Fourth Workshop on Multimedia Signal Processing (Cat. No. 01TH8564).

[81] Wang D., Sun S., Chen X., Yu Z. (2016). A 3D Shape Descriptor Based on Spherical Harmonics Through Evolutionary Optimization. Neurocomputing.

[82] Bellman R. (1966). Dynamic programming. Science.

[83] Yu L., Liu H. Feature selection for high-dimensional data: A fast correlation-based filter solution. Proceedings of the 20th International Conference on Machine Learning (ICML-03).

[84] Kohavi R., John G.H. (1997). Wrappers for feature subset selection. Artif. Intell..

[85] Long F., Peng H., Ding C. (2005). Feature Selection Based on Mutual Information: Criteria of Max-Dependency, Max-Relevance, and Min-Redundancy. IEEE Trans. Pattern Anal. Mach. Intell..

[86] Chen H., Tino P., Yao X. (2009). Probabilistic Classification Vector Machines. IEEE Trans. Neural Netw..

[87] Ertel W. (2011). Introduction to Artificial Intelligence.

[88] Breiman L. (2001). Random Forests. Mach. Learn..

[89] Clark P., Niblett T. (1989). The CN2 Induction Algorithm. Mach. Learn..

