# Supplementary material for: Exploring the Potential of Spherical Harmonics and PCVM for Compounds Activity Prediction
Source: Int J Mol Sci. 2019 May 2;20(9):2175. doi: 10.3390/ijms20092175 (PMC6539940; doi:10.3390/ijms20092175)
Supplement: Supplementary file 1 [file ijms-20-02175-s001.zip › SHPCVM-suppl.pdf]

Supplementary Materials: Exploring the potential of Spherical Harmonics and PCVM for compounds activity prediction

Magdalena Wiercioch

Table S1. The prediction results of PCVM prediction models with a MOE-type descriptor. Scores for the external test set.

UniProt ID	ACC	PRE	REC	MCC	κ
P35372	0.734	0.744	0.711	0.654	0.597
P30542	0.691	0.668	0.703	0.588	0.563
P08908	0.731	0.707	0.724	0.702	0.698
Q9Y5N1	0.713	0.681	0.689	0.601	0.626
Q99705	0.731	0.692	0.702	0.637	0.621
Q14416	0.712	0.678	0.683	0.613	0.591
P21917	0.722	0.681	0.722	0.671	0.646
Q9HC97	0.664	0.648	0.658	0.586	0.573
Q99835	0.732	0.722	0.744	0.684	0.667
P50406	0.695	0.667	0.691	0.648	0.626
Q8TDU6	0.616	0.639	0.609	0.529	0.503
P47871	0.757	0.727	0.772	0.635	0.607
P30968	0.712	0.681	0.666	0.583	0.599
P35348	0.728	0.706	0.712	0.632	0.644
P24530	0.712	0.690	0.725	0.641	0.625
P41180	0.716	0.708	0.724	0.687	0.672
P51677	0.625	0.601	0.636	0.602	0.593
P21452	0.641	0.688	0.668	0.618	0.608
P35346	0.658	0.641	0.662	0.564	0.545
P48039	0.692	0.701	0.703	0.609	0.566
Q9Y5Y4	0.739	0.686	0.742	0.703	0.684

Table S2. The prediction results of PCVM prediction models with a Connectivity descriptor. Scores for the external test set.

UniProt ID	ACC	PRE	REC	MCC	κ
P35372	0.669	0.654	0.678	0.646	0.653
P30542	0.633	0.552	0.581	0.528	0.482
P08908	0.606	0.633	0.614	0.402	0.381
Q9Y5N1	0.607	0.531	0.553	0.488	0.461
Q99705	0.678	0.612	0.626	0.587	0.575
Q14416	0.649	0.591	0.655	0.584	0.571
P21917	0.641	0.627	0.632	0.560	0.552
Q9HC97	0.602	0.581	0.627	0.537	0.563
Q99835	0.669	0.682	0.690	0.632	0.648
P50406	0.592	0.531	0.566	0.481	0.509
Q8TDU6	0.511	0.483	0.507	0.421	0.438
P47871	0.610	0.591	0.628	0.531	0.509
P30968	0.622	0.601	0.616	0.522	0.548
P35348	0.593	0.575	0.593	0.531	0.519
P24530	0.584	0.569	0.598	0.530	0.529
P41180	0.608	0.582	0.593	0.531	0.522
P51677	0.559	0.523	0.548	0.489	0.477
P21452	0.534	0.512	0.549	0.473	0.481
P35346	0.542	0.560	0.531	0.481	0.496
P48039	0.590	0.577	0.599	0.489	0.501
Q9Y5Y4	0.630	0.614	0.649	0.582	0.604

Table S3. The averaged results obtained by cross-validation on the training set in terms of Accuracy.

UniProt ID	PCVM	SVM	RF	NB	*K*NN
P35372	0.835 ± 0.014	0.795 ± 0.012	0.703 ± 0.012	0.664 ± 0.017	0.672 ± 0.016
P30542	0.763 ± 0.012	0.732 ± 0.009	0.652 ± 0.018	0.603 ± 0.019	0.622 ± 0.013
P08908	0.828 ± 0.017	0.772 ± 0.013	0.696 ± 0.017	0.625 ± 0.022	0.658 ± 0.010
Q9Y5N1	0.876 ± 0.014	0.847 ± 0.014	0.762 ± 0.023	0.682 ± 0.020	0.730 ± 0.020
Q99705	0.834 ± 0.014	0.828 ± 0.017	0.726 ± 0.02	0.657 ± 0.016	0.716 ± 0.019
Q14416	0.817 ± 0.015	0.796 ± 0.017	0.703 ± 0.018	0.624 ± 0.016	0.664 ± 0.017
P21917	0.794 ± 0.012	0.744 ± 0.013	0.667 ± 0.013	0.596 ± 0.011	0.614 ± 0.014
Q9HC97	0.805 ± 0.016	0.768 ± 0.012	0.682 ± 0.019	0.623 ± 0.018	0.651 ± 0.016
Q99835	0.863 ± 0.013	0.835 ± 0.014	0.754 ± 0.012	0.691 ± 0.016	0.713 ± 0.014
P50406	0.852 ± 0.014	0.816 ± 0.016	0.725 ± 0.014	0.610 ± 0.011	0.649 ± 0.016
Q8TDU6	0.863 ± 0.017	0.845 ± 0.010	0.757 ± 0.017	0.711 ± 0.009	0.724 ± 0.013
P47871	0.862 ± 0.012	0.832 ± 0.031	0.713 ± 0.013	0.664 ± 0.012	0.653 ± 0.014
P30968	0.827 ± 0.016	0.797 ± 0.014	0.691 ± 0.014	0.610 ± 0.013	0.674 ± 0.018
P35348	0.845 ± 0.013	0.810 ± 0.013	0.796 ± 0.017	0.704 ± 0.014	0.756 ± 0.012
P24530	0.853 ± 0.013	0.824 ± 0.012	0.756 ± 0.011	0.699 ± 0.014	0.734 ± 0.012
P41180	0.867 ± 0.013	0.838 ± 0.012	0.743 ± 0.013	0.667 ± 0.014	0.693 ± 0.016
P51677	0.825 ± 0.012	0.796 ± 0.017	0.684 ± 0.013	0.615 ± 0.017	0.652 ± 0.016
P21452	0.831 ± 0.012	0.791 ± 0.012	0.707 ± 0.012	0.643 ± 0.020	0.654 ± 0.018
P35346	0.794 ± 0.010	0.764 ± 0.015	0.695 ± 0.016	0.622 ± 0.016	0.630 ± 0.021
P48039	0.817 ± 0.011	0.782 ± 0.014	0.705 ± 0.012	0.636 ± 0.015	0.676 ± 0.014
Q9Y5Y4	0.845 ± 0.014	0.792 ± 0.015	0.711 ± 0.014	0.641 ± 0.016	0.683 ± 0.016

Table S4. The averaged results obtained by cross-validation on the training set in terms of Precision.

UniProt ID	PCVM	SVM	RF	NB	*K*NN
P35372	0.832 ± 0.026	0.784 ± 0.021	0.673 ± 0.017	0.636 ± 0.014	0.601 ± 0.015
P30542	0.743 ± 0.020	0.717 ± 0.019	0.627 ± 0.010	0.553 ± 0.013	0.505 ± 0.012
P08908	0.832 ± 0.012	0.784 ± 0.021	0.690 ± 0.009	0.642 ± 0.017	0.603 ± 0.013
Q9Y5N1	0.900 ± 0.014	0.871 ± 0.020	0.736 ± 0.018	0.652 ± 0.020	0.693 ± 0.017
Q99705	0.862 ± 0.016	0.835 ± 0.018	0.713 ± 0.016	0.639 ± 0.013	0.680 ± 0.015
Q14416	0.817 ± 0.018	0.793 ± 0.021	0.603 ± 0.016	0.557 ± 0.012	0.584 ± 0.012
P21917	0.753 ± 0.015	0.703 ± 0.013	0.626 ± 0.013	0.533 ± 0.016	0.594 ± 0.012
Q9HC97	0.784 ± 0.017	0.760 ± 0.016	0.684 ± 0.018	0.522 ± 0.015	0.654 ± 0.015
Q99835	0.891 ± 0.019	0.853 ± 0.020	0.726 ± 0.017	0.631 ± 0.011	0.707 ± 0.014
P50406	0.845 ± 0.019	0.813 ± 0.017	0.707 ± 0.017	0.585 ± 0.015	0.665 ± 0.015
Q8TDU6	0.841 ± 0.010	0.815 ± 0.013	0.682 ± 0.011	0.553 ± 0.014	0.641 ± 0.013
P47871	0.845 ± 0.013	0.816 ± 0.012	0.710 ± 0.012	0.587 ± 0.012	0.649 ± 0.011
P30968	0.835 ± 0.015	0.784 ± 0.012	0.645 ± 0.009	0.585 ± 0.015	0.633 ± 0.014
P35348	0.835 ± 0.010	0.721 ± 0.014	0.696 ± 0.014	0.614 ± 0.015	0.655 ± 0.012
P24530	0.835 ± 0.02	0.811 ± 0.016	0.714 ± 0.014	0.612 ± 0.016	0.661 ± 0.014
P41180	0.890 ± 0.021	0.857 ± 0.018	0.711 ± 0.017	0.629 ± 0.011	0.645 ± 0.015
P51677	0.824 ± 0.015	0.805 ± 0.015	0.694 ± 0.016	0.612 ± 0.013	0.654 ± 0.015
P21452	0.816 ± 0.014	0.773 ± 0.014	0.658 ± 0.014	0.565 ± 0.012	0.651 ± 0.018
P35346	0.822 ± 0.012	0.803 ± 0.013	0.704 ± 0.016	0.613 ± 0.013	0.663 ± 0.016
P48039	0.811 ± 0.018	0.782 ± 0.020	0.655 ± 0.013	0.592 ± 0.015	0.646 ± 0.012
Q9Y5Y4	0.839 ± 0.013	0.784 ± 0.016	0.708 ± 0.012	0.637 ± 0.009	0.678 ± 0.015

Table S5. The averaged results obtained by cross-validation on the training set in terms of Recall.

UniProt ID	PCVM	SVM	RF	NB	*K*NN
P35372	0.844 ± 0.020	0.805 ± 0.022	0.642 ± 0.013	0.604 ± 0.018	0.611 ± 0.016
P30542	0.775 ± 0.020	0.746 ± 0.022	0.646 ± 0.017	0.505 ± 0.021	0.465 ± 0.016
P08908	0.825 ± 0.013	0.761 ± 0.015	0.654 ± 0.017	0.652 ± 0.015	0.584 ± 0.021
Q9Y5N1	0.882 ± 0.022	0.841 ± 0.020	0.663 ± 0.015	0.629 ± 0.016	0.684 ± 0.017
Q99705	0.836 ± 0.021	0.830 ± 0.021	0.659 ± 0.015	0.717 ± 0.015	0.721 ± 0.012
Q14416	0.831 ± 0.019	0.819 ± 0.018	0.587 ± 0.020	0.535 ± 0.018	0.566 ± 0.021
P21917	0.785 ± 0.017	0.735 ± 0.020	0.618 ± 0.017	0.505 ± 0.015	0.608 ± 0.015
Q9HC97	0.807 ± 0.019	0.778 ± 0.016	0.665 ± 0.017	0.510 ± 0.016	0.644 ± 0.018
Q99835	0.843 ± 0.021	0.816 ± 0.021	0.665 ± 0.022	0.604 ± 0.018	0.663 ± 0.019
P50406	0.814 ± 0.020	0.785 ± 0.021	0.673 ± 0.016	0.565 ± 0.018	0.643 ± 0.018
Q8TDU6	0.864 ± 0.014	0.840 ± 0.015	0.655 ± 0.017	0.540 ± 0.017	0.610 ± 0.018
P47871	0.873 ± 0.016	0.842 ± 0.016	0.678 ± 0.016	0.562 ± 0.017	0.664 ± 0.016
P30968	0.857 ± 0.011	0.825 ± 0.013	0.641 ± 0.015	0.629 ± 0.024	0.620 ± 0.020
P35348	0.874 ± 0.017	0.839 ± 0.014	0.669 ± 0.019	0.605 ± 0.014	0.611 ± 0.016
P24530	0.883 ± 0.021	0.855 ± 0.019	0.664 ± 0.022	0.584 ± 0.019	0.612 ± 0.018
P41180	0.835 ± 0.019	0.815 ± 0.018	0.677 ± 0.020	0.603 ± 0.021	0.608 ± 0.017
P51677	0.848 ± 0.017	0.816 ± 0.016	0.671 ± 0.019	0.505 ± 0.021	0.641 ± 0.018
P21452	0.843 ± 0.016	0.819 ± 0.014	0.612 ± 0.015	0.502 ± 0.017	0.612 ± 0.018
P35346	0.794 ± 0.017	0.763 ± 0.019	0.672 ± 0.019	0.562 ± 0.016	0.612 ± 0.021
P48039	0.833 ± 0.014	0.811 ± 0.016	0.615 ± 0.016	0.593 ± 0.020	0.622 ± 0.015
Q9Y5Y4	0.869 ± 0.015	0.818 ± 0.017	0.644 ± 0.016	0.625 ± 0.016	0.647 ± 0.016

Table S6. The averaged results obtained by cross-validation on the training set in terms of Matthews Correlation Coefficient.

UniProt ID	PCVM	SVM	RF	NB	*K*NN
P35372	0.786 ± 0.020	0.746 ± 0.018	0.606 ± 0.024	0.592 ± 0.0217	0.585 ± 0.015
P30542	0.710 ± 0.015	0.677 ± 0.017	0.638 ± 0.020	0.569 ± 0.017	0.408 ± 0.015
P08908	0.776 ± 0.021	0.721 ± 0.014	0.649 ± 0.015	0.615 ± 0.018	0.561 ± 0.018
Q9Y5N1	0.784 ± 0.017	0.762 ± 0.016	0.633 ± 0.019	0.607 ± 0.019	0.632 ± 0.016
Q99705	0.786 ± 0.021	0.759 ± 0.022	0.627 ± 0.020	0.598 ± 0.016	0.617 ± 0.017
Q14416	0.734 ± 0.019	0.719 ± 0.019	0.567 ± 0.017	0.509 ± 0.018	0.524 ± 0.019
P21917	0.806 ± 0.017	0.757 ± 0.018	0.608 ± 0.019	0.464 ± 0.017	0.582 ± 0.016
Q9HC97	0.715 ± 0.018	0.685 ± 0.016	0.629 ± 0.020	0.477 ± 0.018	0.631 ± 0.014
Q99835	0.775 ± 0.013	0.753 ± 0.014	0.652 ± 0.017	0.605 ± 0.0219	0.631 ± 0.014
P50406	0.798 ± 0.017	0.772 ± 0.016	0.661 ± 0.019	0.535 ± 0.018	0.612 ± 0.019
Q8TDU6	0.799 ± 0.015	0.771 ± 0.015	0.629 ± 0.016	0.512 ± 0.017	0.574 ± 0.018
P47871	0.817 ± 0.017	0.792 ± 0.017	0.646 ± 0.018	0.535 ± 0.016	0.632 ± 0.017
P30968	0.795 ± 0.019	0.765 ± 0.019	0.596 ± 0.013	0.603 ± 0.019	0.568 ± 0.018
P35348	0.792 ± 0.012	0.751 ± 0.015	0.627 ± 0.018	0.556 ± 0.015	0.583 ± 0.015
P24530	0.811 ± 0.017	0.775 ± 0.016	0.622 ± 0.020	0.564 ± 0.018	0.585 ± 0.017
P41180	0.808 ± 0.019	0.776 ± 0.019	0.645 ± 0.014	0.585 ± 0.018	0.574 ± 0.017
P51677	0.775 ± 0.018	0.748 ± 0.016	0.616 ± 0.018	0.475 ± 0.017	0.607 ± 0.016
P21452	0.784 ± 0.013	0.745 ± 0.015	0.557 ± 0.017	0.463 ± 0.016	0.578 ± 0.016
P35346	0.716 ± 0.016	0.688 ± 0.016	0.632 ± 0.017	0.545 ± 0.018	0.586 ± 0.019
P48039	0.765 ± 0.017	0.741 ± 0.018	0.605 ± 0.016	0.581 ± 0.017	0.605 ± 0.014
Q9Y5Y4	0.776 ± 0.011	0.725 ± 0.012	0.623 ± 0.015	0.612 ± 0.013	0.617 ± 0.016

Table S7. The averaged results obtained by cross-validation on the training set in terms of κ.

UniProt ID	PCVM	SVM	RF	NB	*K*NN
P35372	0.746 ± 0.018	0.706 ± 0.014	0.616 ± 0.019	0.567 ± 0.019	0.565 ± 0.015
P30542	0.671 ± 0.011	0.638 ± 0.010	0.613 ± 0.019	0.579 ± 0.018	0.381 ± 0.016
P08908	0.771 ± 0.016	0.720 ± 0.017	0.633 ± 0.015	0.641 ± 0.018	0.563 ± 0.018
Q9Y5N1	0.760 ± 0.013	0.707 ± 0.015	0.607 ± 0.018	0.584 ± 0.017	0.607 ± 0.017
Q99705	0.771 ± 0.016	0.721 ± 0.015	0.618 ± 0.020	0.578 ± 0.017	0.617 ± 0.017
Q14416	0.706 ± 0.021	0.679 ± 0.019	0.554 ± 0.018	0.488 ± 0.017	0.539 ± 0.018
P21917	0.795 ± 0.013	0.745 ± 0.013	0.615 ± 0.017	0.484 ± 0.018	0.556 ± 0.016
Q9HC97	0.685 ± 0.015	0.657 ± 0.015	0.610 ± 0.018	0.469 ± 0.014	0.605 ± 0.016
Q99835	0.758 ± 0.010	0.725 ± 0.012	0.635 ± 0.017	0.632 ± 0.019	0.606 ± 0.013
P50406	0.791 ± 0.015	0.757 ± 0.017	0.634 ± 0.015	0.524 ± 0.015	0.595 ± 0.016
Q8TDU6	0.791 ± 0.017	0.755 ± 0.018	0.620 ± 0.015	0.519 ± 0.018	0.556 ± 0.016
P47871	0.813 ± 0.014	0.779 ± 0.014	0.628 ± 0.017	0.546 ± 0.018	0.638 ± 0.018
P30968	0.789 ± 0.015	0.756 ± 0.015	0.575 ± 0.015	0.612 ± 0.017	0.579 ± 0.019
P35348	0.775 ± 0.011	0.739 ± 0.019	0.634 ± 0.017	0.535 ± 0.016	0.596 ± 0.015
P24530	0.776 ± 0.015	0.746 ± 0.013	0.598 ± 0.020	0.556 ± 0.016	0.610 ± 0.017
P41180	0.794 ± 0.015	0.765 ± 0.017	0.636 ± 0.015	0.595 ± 0.016	0.555 ± 0.016
P51677	0.756 ± 0.017	0.714 ± 0.016	0.596 ± 0.018	0.455 ± 0.017	0.594 ± 0.016
P21452	0.747 ± 0.014	0.712 ± 0.015	0.536 ± 0.015	0.445 ± 0.017	0.565 ± 0.014
P35346	0.684 ± 0.016	0.657 ± 0.017	0.617 ± 0.018	0.534 ± 0.014	0.568 ± 0.017
P48039	0.738 ± 0.012	0.703 ± 0.013	0.592 ± 0.017	0.564 ± 0.015	0.576 ± 0.017
Q9Y5Y4	0.763 ± 0.014	0.715 ± 0.015	0.607 ± 0.016	0.628 ± 0.014	0.622 ± 0.018
