# Supplementary figures and images for: Exploring the Potential of Spherical Harmonics and PCVM for Compounds Activity Prediction
Source: Int J Mol Sci. 2019 May 2;20(9):2175. doi: 10.3390/ijms20092175 (PMC6539940; doi:10.3390/ijms20092175)

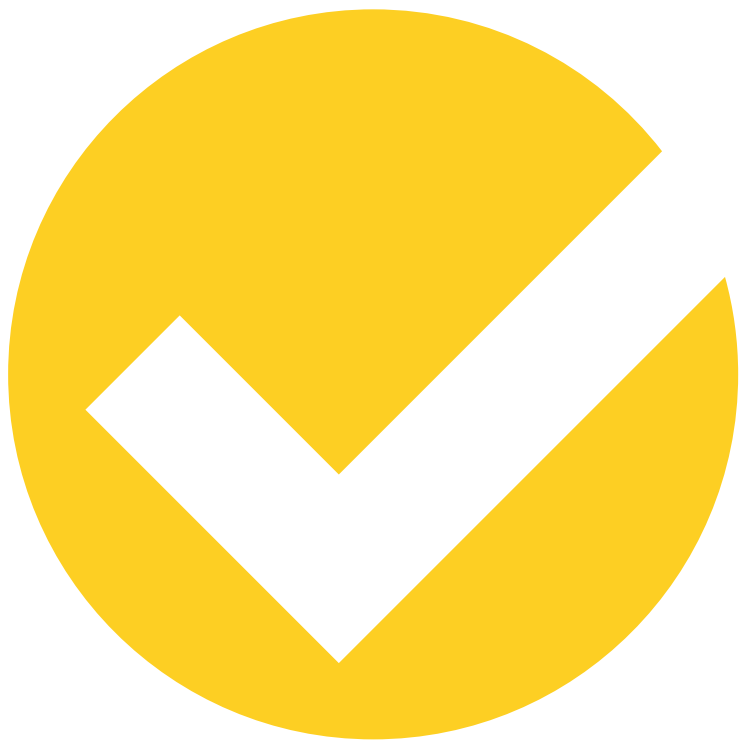

check for  
updates

Supplement: Supplementary file 1 [file ijms-20-02175-s001.zip › Definitions/logo-updates.pdf]
